# Watersports Inclusion Games: The Benefits for Participants and the Impact of COVID-19 on Access

**DOI:** 10.1192/j.eurpsy.2024.973

**Published:** 2024-08-27

**Authors:** M. Killalea, A. Warraitch, J. Murphy, E. Barrett

**Affiliations:** ^1^School of Medicine, University College Dublin; ^2^Irish Sailing; ^3^Child and Adolescent Liaison Psychiatry, Children’s University Hospital Temple Street, Dublin, Ireland

## Abstract

**Introduction:**

The Watersports Inclusion Games is a free annual weekend event, where young people with a range of physical and intellectual disabilities and their families/siblings participate in various inclusive watersports activities.

**Objectives:**

This study aims to assess the psychological benefits of watersports for young people with various physical and intellectual disabilities and investigate the extent of the impact of the COVID-19 pandemic on their access to watersports.

**Methods:**

Following a literature review, a survey containing both quantitative and qualitative aspects was constructed using SurveyMonkey and circulated to the parents/guardians of participants three times following the event. The survey was completed anonymously on an opt-in basis and 28 responses that met our criteria for analysis were collected. Qualitative data from free-text responses were grouped under themes and quantitative data was analysed using SPSS.

**Results:**

Despite 64% (n=18) of respondents indicating that their disability increased their vulnerability to COVID-19 in some capacity, the effect of the pandemic on accessibility was not statistically significant. This could be due to the small response number, or the everyday limitations participants faced prior to the pandemic. 92% (n=25) of participants indicated that there was great inclusion in the watersports activities and that they were “very beneficial” regarding the possibility of the whole family’s participation [p=0.005]. The survey also found a statistically significant association between the event’s activities being considered both “accessible” and “very beneficial” in terms of boosting self-confidence, with 57.1% of responses indicating agreement to this. (p=0.016)

**Conclusions:**

Full-family participation and accessibility of activities were key facilitators to the enjoyment and benefit of participants. Programmes should be established that allow able-bodied siblings and young people with disabilities to participate in the same activities.

**Disclosure of Interest:**

None Declared

